# Enhancement of the Topical Bioavailability and Skin Whitening Effect of Genistein by Using Microemulsions as Drug Delivery Carriers

**DOI:** 10.3390/ph14121233

**Published:** 2021-11-27

**Authors:** Quoc Lam Vu, Chih-Wun Fang, Muhammad Suhail, Pao-Chu Wu

**Affiliations:** 1 School of Pharmacy, Kaohsiung Medical University, 100 Shih-Chuan 1st Road, Kaohsiung City 80708, Taiwan; vuquoclam39@gmail.com (Q.L.V.); suhailpharmacist26@gmail.com (M.S.); 2 Department of Clinical Pharmacy, Thai Nguyen University of Medicine and Pharmacy, 284 Luong Ngoc Quyen Str., Thai Nguyen City 24000, Vietnam; 3 Divison of Pharmacy, Zuoying Branch of Kaohsiung Armed Forces General Hospital, Kaohsiung City 81342, Taiwan; u101530009@gap.kmu.edu.tw; 4 Department of Medical Research, Kaohsiung Medical University Hospital, Kaohsiung 80708, Taiwan; 5 Drug Development and Value Creation Research Center, Kaohsiung Medical University, Kaohsiung 80708, Taiwan

**Keywords:** genistein, microemulsion, topical application

## Abstract

Genistein, the most abundant isoflavone of the soy-derived phytoestrogen compounds, is a potent antioxidant and inhibitor of tyrosine kinase, which can inhibit UVB-induced skin carcinogenesis in hairless mice and UVB-induced erythema on human skin. In current study, genistein-loaded microemulsions were developed by using the various compositions of oil, surfactants, and co-surfactants and used as a drug delivery carrier to improve the solubility, peremability, skin whitening, and bioavailbility of genistein. The mean droplet size and polydispersity index of all formulations was less than 100 nm and 0.26 and demonstrated the formation of microemulsions. Similarly, various studies, such as permeation, drug skin deposition, pharmacokinetics, skin whitening test, skin irritation, and stability, were also conducted. The permeability of genistein was significantly affected by the composition of microemulsion formulation, particular surfactnat, and cosurfactant. In-vitro permeation study revealed that both permeation rate and deposition amount in skin were significantly increased from 0.27 μg/cm^2^·h up to 20.00 μg/cm^2^·h and 4.90 up to 53.52 μg/cm^2^, respectively. In in-vivo whitening test, the change in luminosity index (ΔL*), tended to decrease after topical application of genistein-loaded microemulsion. The bioavailability was increased 10-fold by topical administration of drug-loaded microemulsion. Conclusively, the prepared microemulsion has been enhanced the bioavailability of genistein and could be used for clinical purposes.

## 1. Introduction

Genistein (4′,5,7-trihydroxyisoflavone), a heterocyclic diphenol with three hydroxyl groups, is the main isoflavone found in soybeans and has various biological effects, including antimicrobial, anti-inflammatory, antioxidant, chemoprevention, and enhancement of menopausal symptoms [1,2,3,4,5]. In terms of toxicity, genistein is not mutagenic to Salmonella typhimurium and is not mutagenic or teratogenic to mice and rats. However, genistein has a topoisomerase II inhibitory effect, which may cause chromosomal damage with a threshold dose response [6,7,8]. In dermatology, genistein has proven with potent antiphotoaging and antiphotocarcinogenic effects. Its possible mechanisms are scavenging of reactive oxygen species (ROS), hindering photodynamic DNA damage, downregulation of the phosphorylation of receptor epidermal growth factor receptor (EGF-R), reduction of the activation of mitogen-activated protein kinase (MAPK), and suppression of oncoprotein expression and inhibition of tyrosine protein kinase [9,10,11]. In addition, topical application of genistein can effectively protect mouse skin against photodamage induced by psoralen plus UVA (PUVA) and block the UVB-induced erythema in human skin by dose dependency [10]. However, genistein is a lipophilic compound (Log P = 3.04), and its solubility in water is low (5.3 μM) at 25 °C; therefore, it is difficult to transport across the skin membrane [12], which limits it topical application. Hence, pharmaceutical technology is needed to overcome these obstacles.

As early as the 1940s, Hoar and Schulman produced a clear, single-phase solution by titration, a milky emulsion containing hexanol, and named it a microemulsion [13]. Nowadays, it is defined as a homogeneous, thermodynamically stable transparent colloid with a low viscosity and nanoscale droplet size (generally several hundred nm), which is composed of water and oil and stabilized by a mixture of surfactant and co-surfactant (typically an alkyl alcohol). Microemulsion has many advantages, including clarity, ease of preparation (spontaneous emulsification), long-term stability, increase of solubility of the therapeutic compounds, and enhancement of bioavailability of the hydrophobic drugs by maintaining them in molecular dispersion; therefore, it has been widely used as a pharmaceutical drug delivery carrier, including oral [14,15], ocular [16,17], transdermal/topical [18,19], nasal [20,21], vaginal [22,23], pulmonary [24], intravesical [25,26], and parenteral drug delivery systems [27]. Due to microemulsion’s favorable physicochemical properties (low viscosity and nanoscale droplet size), the role of penetration enhancer played by its amphiphilic components, and less irritation with skin, it is thus considered as a promising drug carrier for transdermal drug delivery system [28,29,30,31]. Therefore, the designed microemulsion system was used as a drug carrier (vehicle) to enhance the drug solubility and permeability of genistein through topical application. In this study, the effect of microemulsion composition, including oil, surfactant, and cosurfactant, on solubility and permeability were investigated. The effect of skin whitening, bioavailability, irritation, and stability of genistein-loaded formulation were also determination to evaluate the clinical utility.

## 2. Results

### 2.1. Solubility in Different Vehicles

The drug solubility in different vehicles is listed in Table 1. Genistein is a hydrophobic compound, and its solubility is about 0.01 mg/mL in distilled water. The solubility in oil, surfactant, and co-surfactant increased by more than 13-fold, 1903-fold and 8415-fold, respectively. The oil of Capryol 90 showed highest solubility; hence, it was used as oil. In surfactants, labrasol showed highest solubility, followed by Brij 30, Cremophor, and Tween 80. In cosurfactants, the drug solubility in Transcutol HP was higher than that of PEG 400.

### 2.2. Characterizations of Formulations

Microemulsion is a clear, thermodynamically stable isotropic liquid mixture of oil, water, and surfactant frequently in combination with a cosurfactant. Surfactant is the key components for the microemulsion formulations design, as it can form a film at the interface between the oily and aqueous phases, which leads to the reduction of interfacial tension and consequently spontaneous formation of stable microemulsion formulations [25,32]. Nonionic surfactants are widely used in pharmaceutical formulation due to their low toxicity, low irritancy, high tolerance, and high compatibility as compared ionic surfactants. Therefore, nonionic surfactant of Tween 80 (T80), Cremophor EL (CEL) and Labrasol (LAB) were used to prepare drug-loaded microemulsions and to evaluate the effect on drug permeability as well as characterization of the formulations. The viscosity, mean droplet size, and polydispersity index of genistein-loaded microemulsion formulations are listed in Table 2. The mean droplet size and polydispersity index of all formulations was less than 100 nm and 0.26, demonstrated the formation of microemulsions with low viscosity by the use of these surfactant and proportion.

### 2.3. Drug Release and Skin Permeation

**Figure 1:**
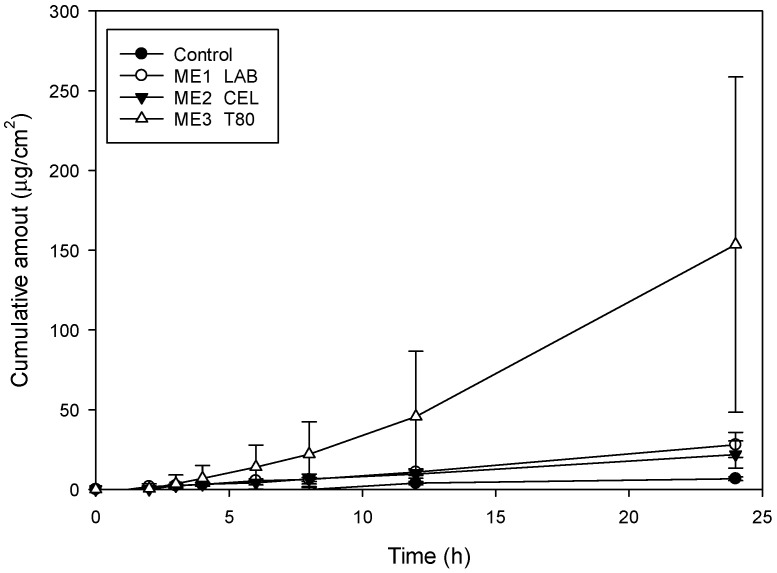
The permeation profiles of genistein dissolved in 30% ethanol (control) and microemulsions through rat skin (*n* = 3).

The permeation profiles of genistein-loaded microemulsion formulation and drug in 30% ethanol solution (control group) microemulsions through the skin are plotted in Figure 1, and permeation parameters are summarized in Table 2. In control group, it can be seen that genistein is not easily transported through the skin; the lag time (first detected time) was about 24 h. The permeation rate (flux) and deposition amount in skin was 0.27 μg/cm^2^·h and 4.90 μg/cm^2^, respectively. In term of microemulsion groups, the permeability of the genistein through skin was significantly improved. The flux and deposition amount in skin were increased up to 6.46 μg/cm^2^·h and 21.86 μg/cm^2^, about a 23.9-fold and 4.5-fold increase, respectively. The lag time was shortened to 5.67 h. The result was agreed with previous research results, which reported that microemulsion system could improve permeability of drug [29,30,33]. The possible enhancement mechanism of the microemulsion system might be attributed to the following: (1) the components of surfactant and cosurfactant could act as permeation enhancers; (2) the nano-scale droplet size could offer high hydration of skin layer; and (3) lower viscosity and smaller droplet size exhibited higher permeability [19,34,35,36,37]. In this study, the formulation containing Tween 80 showed highest flux and shortest lag time. There is no significant difference in the deposition amount in skin of the three groups of formulas.

**Figure 2:**
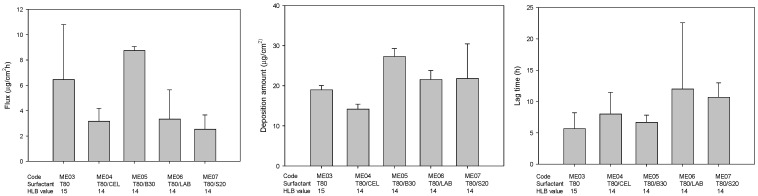
The effect of different kinds of mixture surfactants on the permeation parameters of genistein-loaded microemulsions (*n* = 3) (T80, Tween 80; B30, Brij 30; LAB, Labrasol; S20, Span20).

Previous studies reported that it is easier to form a more stable microemulsion when mixed with hydrophilic and hydrophobic surfactants [38,39]. Moreover, the permeation ability of microemulsion could be enhanced when lower HLB value of surfactant was incorporated because it could provide better interaction with the skin layers [40,41]. Hence, the effect of different kind and HLB of mixture surfactants on permeability were evaluated in this study, and the drug permeation parameters, including flux, drug deposition amount in skin, and lag time, are plotted in Figure 2. It was found that formulation containing mixture surfactant Tween 80 and Brij 30 showed higher flux, higher disposition amount, and lower lag time compared to that of formulation containing only Tween 80, indicating that an appropriate combination was necessary for genistein transdermal delivery. Similarly, previous studies reported that the right blend of low- and high-HLB surfactants is necessary for the formation of a stable microemulsion because of mixture surfactant with two extreme HLB values would result in a strongly hydrophobic surfactant that can dissolve mostly in the oil phase, with the strongly hydrophilic surfactant conversely dissolving mostly in the water phase and then strengthening the stability of the oil-water interfacial film [42,43,44].

**Figure 3:**
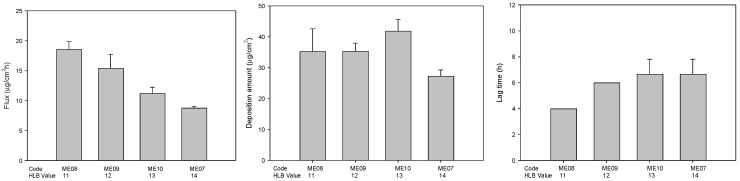
The effect of different HLB value of mixture surfactants on the permeation parameters of genistein-loaded microemulsions (*n* = 3).

The HLB value of mixture surfactant also plays an important role in the determination of formation of the stable microemulsions and drug delivery through the microemulsions. Therefore, effect of microemulsions containing Tween 80 and Brij 30 with different HLB value was evaluated. As shown in Figure 3, the flux gradually decreased when the HLB value of mixed surfactants in the formulations increased. The phenomena might be attributed to the high HLB values result in a reduction in surface free energy [40].

**Figure 4:**
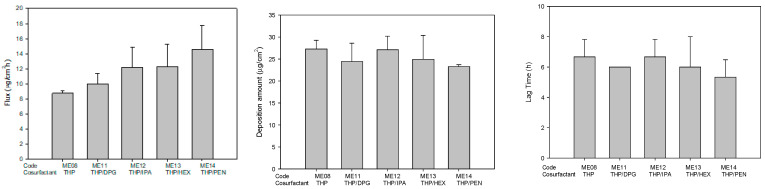
The permeation parameters of genistein-loaded microemulsions containing single and mixture cosurfactants. (THP, Transcutol HP; DPG, Dipropylene Glycol; IPA, isopropyl alcohol; HEX, 1,2-hexanediol; PEN, 1,5-pentanediol) (*n* = 3).

**Figure 5:**
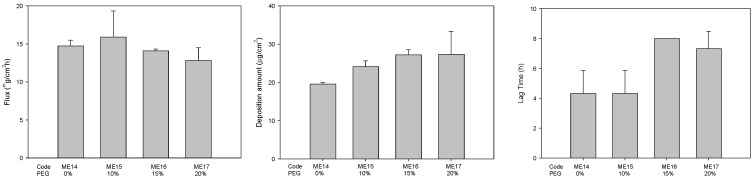
The effect of amount PEG (0~20%) on the permeation parameters of genistein-loaded microemulsions (*n* = 3).

The use of mixtures of components, such as surfactants or cosurfactants, is an interesting approach from the pharmaceutical point of view since the use of mixtures allows the individual concentration of each component to be decreased, which may increase the biocompatibility of the final formulations [38,45,46]. Therefore, the 10% THP in formulation was replaced with other co-surfactants, including IPA, DPG, HEX, and PEN, and the effects on the permeability were evaluated. The effect of mixture cosurfactant on drug permeability was also evaluated in this study. As shown in Figure 4, the microemulsion containing mixture cosurfactant THP/PEN had highest flux. In order to dissolve more hydrophobic drug genistein, PEG 400 was added to the microemulsion. When PEG 400 concentration was increased from 0 to 20%, the solubility was increased from 11.08 mg/mL to 32.01 mg/mL. The flux was decreased slightly, whereas the drug deposition amount and lag time were increased (Figure 5).

**Figure 6:**
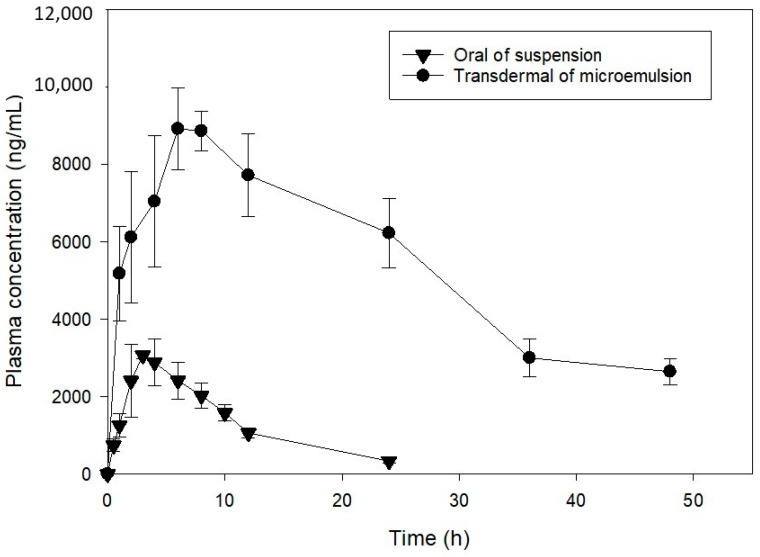
Mean plasma concentration-time curve (mean ± S.D., *n* = 3) of genistein after oral administration of suspension and transdermal administration of microemulsion.

The pharmacokinetic evaluation of the topical application of genistein-loaded microemulsion (ME15) with higher permeability and oral administration of drug aqueous suspension was conducted. The plasma concentration of genistein plotted against time after oral and topical administration at a dose of 20 mg/kg is illustrated in Figure 6, and the pharmacokinetic parameters are shown in Table 3. The genistein concentration in plasma after oral administration was rapidly decreased because of being extensively metabolized and excreted [47,48,49]. The Cmax, AUC_0__→__ꝏ_, and t_1/2_ were 3.12 ± 1.80 µg/mL, 33.9 ± 4.76 µg/mL·h, and 6.52 ± 1.24 h, respectively. The result was similar to the previous report [49]. As expected, the plasma concentration of genistein could be maintained at high levels for a long time after transdermal application of drug-loaded formulation. The Cmax increased up to 9.12 ± 0.57 µg/mL, about a 3-fold increase. The AUC_0__→__ꝏ_ was significantly increased from 33.91 ± 4.76 µg/mL·h to 329.30 ± 50.03 µg/mL·h, approximately a 10-fold increase. This phenomenon might be due to the metabolization of genistein in the intestine [50]. The relative bioavailability of transdermal administration was significantly increased about 10-fold compared to oral administration.

### 2.4. Skin Whitening Effect

**Figure 7:**
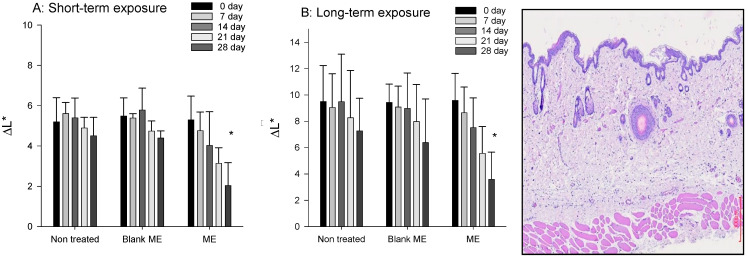
The change degree of luminosity index (ΔL*, whitening effect) of genistein-loaded microemulsion after UV induced hyperpigmentation. (**A**) short-term exposure; (**B**) long-term exposure (*n* = 6).

**Figure 8:**
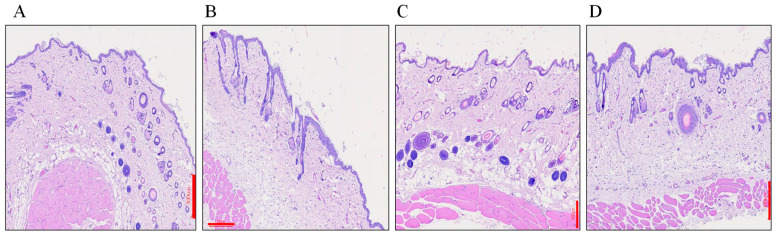
The photomicrographs of rat skin section. (**A**) Distilled-water-treated skin, (**B**) 0.8% formalin solution-treated, (**C**) drug-free formulation treated, and (**D**) genistein-loaded formulation treated. Scale bar = 300 μm.

The skin whitening effect was conducted for the purpose to determine the change degree of luminosity index (ΔL*) after 28 days of daily topical application of samples (Figure 7). For the short-term exposure (inhibition of tyrosinase) [51], the values of ΔL* of non-treated group and blank formulation-treated group were slightly decreased time dependently, whereas there was significant decrease at 21 and 28 days of drug-loaded formulation treated group. Previous study reported that genistein has inhibition effect of tyrosinase [2,9,10]. The whitening effect demonstrated that genistein was transported into the skin by the microemulsion carrier. For the long-term exposure (reduce the melanin production), it still can be observed that values of ΔL* of drug-loaded formulation-treated group were significantly decreased indicated that the melanin production was decreased (Figure 8) [52].

### 2.5. Skin Irritation Evaluation

The irritation test was conducted to assess the safety of tested microemulsion formulations. The distilled water-treated and 0.8% formalin solution-treated were used as negative control and standard irritant group, respectively. As shown in Figure 8A, the tissue, such as SC, epidermis, and dermis layers, were completed and well defined. In contrary, some slight damage and exfoliation of the stratum corneum in the epidermis layer, small edema in the hypodermis layer, and collagen fiber swelling in the dermis layer was observed in the standard irritant group (Figure 8B.) In the tested drug-free formulation (Figure 8C) and genistein-loaded formulation (Figure 8D), non-obvious erythema and edema were found when compared to the negative group, showing that the designed microemulsion formulation possesses good biocompatibility with skin tissue and can be used clinically.

### 2.6. Stability Evaluation

For thermodynamic stable test, no phase separation, turbidity, creaming, or cracking of tested formulation was observed after centrifugation at 10,000 rpm for 5 min at 25 °C and three freeze-thaw cycles between −21 °C and +25 °C. Before test, the droplet size and viscosity were 90.82 ± 1.34 nm and 20.17 ± 0.50 cps, respectively. After test, the droplet size and viscosity were 86.62 ± 1.91 nm and 18.30 ± 0.36 cps for centrifugation test and 93.50 ± 1.74 nm and 17.77 ± 0.25 cps, respectively. There was a non- significant change (*p* > 0.05), indicating thermodynamic stability of the tested genistein-loaded formulations. Previous studies pointed that microemulsion was thermodynamically stable because of low interfacial tension between water phase and oily phases and its nanoscale droplet size [28,33].

After 3 months of storage at 25 ± 2 °C, RH 60 ± 5% and 40 ± 2 °C, RH 75 ± 5%, the appearance of the genistein-loaded formulation showed no obvious change, and no precipitate was found. The residual genistein contents of tested formulations were 93.74 ± 1.23% and 90.24 ± 1.56% at 25 °C and 40 °C storage, respectively, indicating that the tested formulations were stable.

## 3. Materials and Methods

### 3.1. Materials

Genistein, diazepam, dipropylene glycol (DPG), and 1,5-pentanediol (PEN) were acquired from Alfa Aesar (Lancashire, UK). Nicotine and sulfatase were purchased from Sigma-Aldrich (St. Louis, MI, USA). Capryol 90 (CAP), Transcutol HP (THP), and Labrasol (LAB, hydrophilic lipophilic balance (HLB) =14) were obtained from Gattefosse (Courbevoie, France). Span 20 (S20, HLB = 8.6) was purchased from Tokyo Chemical Industry (Tokyo, Japan); Tween 80 (T80, HLB = 15) was obtained from Showa Corporation (Japan); Cremophor EL (CEL, HLB = 12~14) was purchased from Fluka (Munich, ND, USA). Brij 30 (B30, HLB = 9.5) was purchased from Acros organic (Waltham, MA, USA); propylene glycol (PG), isopropyl alcohol (IPA), and polyethylene glycol 400 (PEG) were purchased from Merck Chemicals (Gernsheim, Germany). All other chemicals and solvents were of analytical reagent grade.

### 3.2. Solubility of Genistein in Differernt Vehicles Determination

An excess amount of genistein was added to 1 mL each of the vehicles, including receptor buffer and oils, into a 1.5-mL Eppendorf, which was placed on a reciproacting shaker (Shaking Bath, Model B601D, Firstek Co., Ltd., Taipei, Taiwan) and shaken for more than 24 h at room temperature. Then each smaple was centrifuged at 12,000 rpm for 10 min. The drug concentration in supernatant was analyzed by HPLC.

Hitachi HPLC system (Hitachi, Tokyo, Japan), including model L-7100 pump, L-5210 autosampler, model L-4000H detector, and Merck Lichrocart^®^ 100 RP 18 column (250 mm × 4 mm, 5 μm), was used. The mobile phase was composed of methanol and 0.05% phosphoric acid aqueous solution at ratios of 80/20 *v*/*v*. The flow rate and detection wavelength were at 1.0 mL/min and 230 nm, respectively. Diazepam solution was used as internal standard. The analytical method was successfully validated for linearity (1.0~100.0 μg/mL), with a determination coefficient (R^2^) of 0.9997, precision of coefficient of variation (CV, %) of 4.16%, and accuracy of relative error (RE, %) of 5.71%. The limit of quantitation was 0.5 µg/mL.

### 3.3. Genistein-Loaded Microemulsions Preparation

The composition of drug-loaded microemulsions (ME1 to ME3) is listed in Table 4. The mixture of surfactants with specific hydrophilic lipophilic balance (HLB) value or ratio was mixed well in advance. Oil phase of Capryol 90, mixture surfactants, and cosurfactant were mixed well by a vortex at room temperature. Then, distilled water was slowly added to the previous mixture while shaking, and the mixture was then vortexed for 1 min to form the transparency and clarity of microemulsion formulations. Genistein of 1%~3% was dissolved in the microemulsion formulations by a horizontal shaker for 24 h. Then visually check the appearance of the formualtions. All durg-loaded microemulsions were clear without any precipitation.

### 3.4. Characterization of Genistein-Loaded Formulation Determination

The average droplet size and polydispersity index of the drug-loaded formulations were determined by a photo correlation spectroscopy equipped with laser light scattering (Malvern Instruments, Ltd., Malvern, UK). The test was conducted in a thermostatic chamber at 25 °C in triplicate.

The viscosity of the drug-loaded formulations was measured at 37 °C, using a cone-plate of viscometer (Brookfield, Model LVDV-II, USA) with stir rate of 100 rpm in triplicate.

### 3.5. Transdermal Permeation Study

All animal experimental protocol (#109027) was confirmed and approved by the Institutional Animal Care and Use Committee of Kaohsiung Medical University (Kaohsiung, Taiwan). Similarly, all experimental procedures were conducted according to the guidelines as set forth by the Guide for Laboratory Fact lines and Care.

In this study, male Sprague–Dawley (SD) rats weighing 260–330 g were used for the purpose to evaluate the transdermal delivery of the tested formulations. Animals were anesthetized by chloral hydrate of 10% (dosing 0.3 mL/100 g) for intraperitoneal injection and humanely sacrifice. The abdominal hairs of the rats were removed with an electric shaver, and the skin of the abdomen was excised. The subcutaneous tissue and fat were completely removed, and the integrity of the skin was verified. The skin samples were subsequently stored at −20 °C until used.

#### 3.5.1. In-Vitro Transdermal Permeation

The in-vitro transdermal permeation experiment was conducted by using a modified Franz diffusion cell through a piece of rat skin. The skin was sandwiched between the donor and receiver cells with dermis layer of the skin toward the receiver side, and the available transdermal area was 3.46 cm^2^. Each receptor cell was loaded 20 mL of pH 7.4 phosphate-buffered saline soltuion contining 20% ethanol as the receptor phase to maintain sink condition and continuously stirred with a magnetic bead at 600 rpm, with a temperature set at 37 ± 0.5 °C during the entire experiment by a water circulation jacket. One mimilitter of tested formulation was applied over the surface of the rat skin. To prevent evaporation of water from the formulations, the donor cell was occluded with Parafilm^®^. At derminal time interval of 0.5, 1, 2, 3, 4, 6, 8, 12, and 24 h, 1 mL of samples was taken from the receptor cell and replenished with same volume of fresh receptor solution. The amount of the genistein transported across the skin was determed by a modified HPLC.

#### 3.5.2. Drug Skin Deposition

At the end of the in-vitro transdermal permeation experiment, the applied rat skin was taken off from the Franz cell, and carefully removed the residual formulation was carefully removed with an absorbable cotton tip. The skin surface was further washed with deionized water for six alternate times to remove excess drug. Then, the skin was cut into small pieces and placed in glass vials containg 4 mL of receptor solution phase, followed by extracting for 12 h with horizontal shaken. After extraction, 1 mL of the extracted solution was collected and centrifuged for 10 min at 3000 rpm, and the supernatant was analyzed by HPLC for evaluation the deposition amount of genistein in rat skin.

The genistein content was determined using HPLC system (Hitachi, Japan) equipped with a model L- 2130 pump, model L- 2200 autosampler, model L-7420 detector, and a C18 column (Lichrocart^®^, 250 mm × 4.6 mm I.D., particle size 5 μm, Merck, German). The detector wavelength was set at 230 nm. A mixture of 0.05% phosphoric acid and methanol (80/20) was used as a mobile phase. Flow rate was kept 1 mL/min. The analytical method was successfully validated for linearity (1–100 μg/mL) with a determination coefficient (R^2^) of 0.9997, coefficient of variation of 4.16%, and relative error of 5.71%. The limit of detection was 0.5 μg/mL. The retention time of genistein and diazepam (internal standard) was 4.1 and 5.8 min, respectively.

#### 3.5.3. Calculation of Transdermal Parameters

The permeation cumulative amounts of genistein were used to calculate the transdermal flux (J, μg/(cm^2^·h)) according to the following equation:

J = dQ/dt·A

where Q (μg) is the permeation cumulative amount of drug (μg), t (h) is the permeation time, and A (cm^2^) is the applied transdermal area of the skin. Lag time (h) is the first detected time of drug (h).

### 3.6. In Vivo Pharmacokinetic Determination

Male Sprague−Dawley (SD) rats weighing 260–310 g and who were 7 weeks old were housed in an air-conditioned room with the temperature maintained at 25 °C ± 1 °C and humidity at 55% ± 5%. Rats were divided into two groups, i.e., tranderaml (test) group and oral (control) group, each of three. Rats were anesthetized by 10% chloral hydrate (dosing 0.3 mL/100 g) for intraperitoneal injection. After being anesthetized, hairs on the abdominal skin were removed by an electric shaver. Then, a glass ring with a diameter of 3 cm was fixed on the skin by a quick-drying glue. The applied dose of 20 mg/kg of the tested microemulsion formulation was placed inside the glass ring. With control group: genistein suspension of 40 mg/mL in distilled water was applied at dose of 20 mg/kg for oral administration. Blood samples of approximately 0.6 mL were collected from the jugular vein and placed into a 1.5-mL Eppendorf at predetermined time intervals after transdermal administration. Then, the blood sample was centrifuged at 12,000 rpm for 10 min. The plasma samples were stored at −20 °C until analysis. Three to six replicates were conducted for each formulation.

Sample preparation [49,53,54]: A 100 μL of plasma samples was incubated with 100 μL of the sulfatase (approximately 200 unit) at 37 ± 0.5 °C for 5 h. Then, 100 µL Nicotine 4 µg/mL was used as internal standard added to the sample, then vortexed for 10 s. A total of 1 mL tert-methyl butyl ether was added for extract solution and vortexed for 10 min and then centrifuged at 10,000 rpm for 10 min. The supernatant of 800 µL was transferred to Eppendorf and evaporated to dryness in the vacuum oven (EYELA CVE-3000, Tokyo, Japan) for 1 h and 40 °C. The residue was reconstituted with 100 µL methanol and analyzed.

The pharmacokinetic analysis includes the following quantities: area under the curve (AUC), maximum plasma concentration (Cmax), the time needed to reach the maximum plasma concentration (Tmax), and the half-life (t_1/2_) of the drug. All values are presented as the mean ± standard deviation (SD). All data were processed using Phoenix WinNonlin 6.3 (Pharsight Corporation, Sunnyvale, CA, USA) to construct pharmacokinetic profiles. Data were analyzed by using the ANOVA test to evaluate the differences between the groups, and values of *p* < 0.05 indicated significant differences compared with control.

### 3.7. In Vivo Skin Whitening Effect Test

#### 3.7.1. Short-Term Exposure Test

Six male guinea pigs (body weight 350–450 g) were used in this study. The hair on the dorsal skin was shaved with an electric shaver for two separate slots (1.5 cm × 1.5 cm). We recorded the Pre-exposure L* (luminosity index represents the level of pigmentation of the skin) by the colorimeter (Minolta CR-221, Tokyo, Japan), which was regarded as the 0th day. Then, the dorsal skin was exposed to UVB irradiation with total irradiation energy of 840 mL/cm^2^ for 14 min and the UVB intensity of 2 mW/cm^2^. After exposure, blank microemulsion (without drug) and drug-loaded microemuliosn at a dose of 20 mg/kg was applied to the allotted regions skin with a micropipette. The application was performed once per day for 28 days after exposure, on weekdays. On 7th, 14th, 21st, and 28th day after exposure, the colorimeter was used to observe and record the L* of reading (nth day) [51,52,55,56,57].

#### 3.7.2. Long-Term Exposure Test

The dorsal skin of guinea pigs was exposed to UVB irradiation, 3 times a week (every other day) for 2 consecutive weeks. The UVB intensity was 2 mW/cm^2^, and the total energy dose was 1 J/cm^2^ per exposure for 10 min. Then the animals were then left for an additional week to allow the UVB induced hyperpigmentation to stabilize. After that, test samples, including blank microemulsion (without drug) and drug-loaded microemulsion, were topically applied daily to the hyperpigmented areas for 28 days at a dose of 20 mg/kg. The degree of pigmentation was assessed as the L* of reading measured with a colorimeter at 7th, 14th, 21st, and 28th day after exposure [52]. The change in luminosity index ΔL* was calculated as:

ΔL* = Pre-exposure L* − L* of reading (nth day)

### 3.8. Skin Irritation Determination

The histological examination method was used to evulate the skin irritation caused by tested formulations. The SD rats were divided into four groups including negative control groups (untreated group), positive control group (treated with 0.8% paraformaldehyde), treated with drug-free formulation group, and drug-loaded formulation treated group, each with 3 guinea pigs. The abdominal hairs of animals were carefully removed by an electrical shaver one day before the skin irritations stest. For testing, 1 mL of the test sample was evenly spread on the shaven abdomen skin of 3.46 cm^2^ and then occluded by parafilm. After 24 h exposure, rats were sacrificed, and the applied skin tissue was excised for histological examination. In brief, the skin tissue was fixed in 4% buffered formaldehyde solution at least for 24 h before routine processing, including rinsing with running distilled water, dehydrating using with a graded series of ethanol solution, and embedding in paraffin. Then, the tissue sample was sliced transversely into 20-μm thickness, rehydrated, and stained with hematoxylin-eosin for histological microscopic observation (Nikon Eclipse Ci, Tokyo, Japan) [33,58].

### 3.9. Stability Study

Thermodynamic stability: tested genestein-loaded formulation was subjected to different stress conditions, such as centrifugation and freeze-thaw cycle. The tested formulation was centrifugated at 10,000 rpm for 10 min and then subjected to three freeze-thaw cycles at temperatures between freeze temperature (−21 °C) and room temperature (25 °C), with storage at each temperature for not less than 48 h to assess the physical stability of test sample [25,59]. The phase separation, change in globule size, and viscosity was examined.

Long-term stability: The tested formulation was stored in dark-brown bottles for protection from light. The stability of formulation was evaluated via clarity and phase separation observation and drug content at 25 ± 2 °C, relative humidity 60 ± 5%, and 40 ± 2 °C relative humidity 75 ± 5%.

## 4. Conclusions

Different formulations of genistein-loaded microemulsions were prepared successfully by the various combinations of oil, surfactant, and co-surfactant. When used microemulsion as a drug delivery carrier, the permeation rate and deposition amount in skin were significantly increased from 0.27 μg/cm^2^·h up to 20.00 μg/cm^2^·h and 4.90 up to 53.52 μg/cm^2^, respectively. The result indicated that formulation factors, such as oil type, the type, HLB value, and combined use of surfactants and cosurfactants, had a significant impact on the solubility and permeability of genistein. Pharmacokinetic study indicated that the relative bioavailability of transdermal administration of genistein was significantly increased by formulated microemulsion about 10-fold compared to oral administration. The whitening effect indicated the transportation of genistein across the skin and revealed a decrease in the production of melanin. Similarly, the skin irritation study evaluated a good compatibility of microemulsion with the skin. In addition, the stability study revealed the good stability of tested formulations after three months of storage. The above results demonstrated that genistein-loaded microemulsions have the potential to enhance the bioavailability of genistein.

## Figures and Tables

**Table 1:** Solubility of genistein in different vehicles.

Vehicles	Solubility (mg/mL)
Capryol 90	5.20 ± 0.23
Peceol	0.36 ± 0.01
Oleic acid	0.13 ± 0.02
Tween 80	19.03 ± 1.37
Labrasol	55.48 ± 3.56
Cremophor EL	19.43 ± 6.15
Brij 30	27.74 ± 1.80
Transcutol HP	94.65 ± 3.92
PEG 400	84.15 ± 5.61
Distilled water	0.01 ± 0.00

**Table 2:** The composition, characteristics, and permeation parameters of genistein-loaded microemulsions with different surfactant.

	Surfactant	Flux (μg/cm^2^·h)	Lag Time (h)	Deposition (μg/cm^2^)	Viscosity (Cps)	Size (nm)	PDI
C		0.27 ± 0.04	24.00 ± 0.00	4.90 ± 1.88			
ME1	LAB	1.17 ± 0.34	8.67 ± 3.06	21.86 ± 3.22	15.80 ± 0.30	71.37 ± 2.74	0.26 ± 0.01
ME2	CEL	0.92 ± 0.37	8.67 ± 3.06	19.57 ± 4.78	6.78 ± 0.20	74.97 ± 0.15	0.09 ± 0.04
ME3	T80	6.46 ± 4.34	5.67 ± 2.52	18.97 ± 1.07	14.27 ± 0.45	49.73 ± 1.63	0.26 ± 0.02

C, control (1% drug dissolved in 30% ethanol); PDI, polydispersity index.

**Table 3:** Pharmacokinetic parameters of genistein following the oral (aqueous suspension) and transdermal (microemulsion) administration of genistein in rats (20 mg/kg) (mean ± S.E., *n*= 3).

Parameters	Oral	Transdermal
C_max_ (µg/mL)	3.12 ± 1.80	9.12 ± 0.57 *
T_max_ (h)	3.67 ± 0.43	7.26 ± 1.11
t_1/2_ (h)	6.52 ± 1.24	19.34 ± 5.05
AUC_0→ꝏ_ (µg/mL·h)	33.91 ± 4.76	329.30 ± 50.03 *

C_max_, peak plasma concentration; T_max_, time to peak concentration; t_1/2_, elimination half-life; AUC, area under the curve. * *p* < 0.05.

**Table 4:** The composition of drug-loaded microemulsions with different surfactants.

	Drug	Oil	Surfactant						HLB	Cosurfactant		Water
Code	GEN	CAP	LAB	CEL	T80	B30	S20			THP		Water
ME01	1.0	5.0	15	-	-	-	-		12	32.0		47.0
ME02	1.0	5.0		15	-	-	-		12	32.0		47.0
ME03	1.0	5.0		-	15	-	-		15	32.0		47.0
ME04	1.0	5.0		7.5	7.5	-	-		14	32.0		47.0
ME05	1.0	5.0		-	12.5	2.5	-		14	32.0		47.0
ME06	1.0	5.0	5.0	-	10.0	-	-		14	32.0		47.0
ME07	1.0	5.0		-	12.7	-	2.3		14	32.0		47.0
ME08	1.0	5.0	-	-	4.3	10.7	-		11	32.0		47.0
ME09	1.0	5.0	-	-	6.8	8.2	-		12	32.0		47.0
ME10	1.0	5.0	-	-	9.5	5.5	-		13	32.0		47.0
	Drug	Oil	Cosurfactant							Surfactant		Water
Code	GEN	CAP	THP	IPA	DPG	HEX	PEN	PEG	HLB	T80	B30	Water
ME11	1.0	5.0	22.0	10.0		-	-	-	11	4.3	10.7	47.0
ME12	1.0	5.0	22.0	-	10.0	-	-	-	11	4.3	10.7	47.0
ME13	1.0	5.0	22.0	-	-	10.0	-	-	11	4.3	10.7	47.0
ME14	1.0	5.0	22.0	-	-	-	10.0	-	11	4.3	10.7	47.0
ME15	1.0	5.0	22.0			-	10.0	10.0	11	4.3	10.7	37.0
ME16	1.0	5.0	22.0			-	10.0	15.0	11	4.3	10.7	32.0
ME17	1.0	5.0	22.0			-	10.0	20.0	11	4.3	10.7	27.0

The total amount of each formulation was 100 g. GEN, Genistein; CAP, Capryol 90; LAB, Labrasol; CEL, Cremophor EL; T80, Tween 80; B30, Brij 30; S20, Span20; THP, Transcutol HP; IPA, isopropyl alcohol; DPG, Dipropylene Glycol; HEX, 1,2-hexanediol; PEN, 1,5-pentanediol; PEG, Polyethylene Glycol 400; HLB, hydrophilic-hydrophobic balance.

## Data Availability

Data is contained in the article.
